# Herpesvirus immunology in solid organ transplant recipients – liver transplant study (HISTORY): a retrospective and prospective observational cohort study

**DOI:** 10.1186/s12879-023-08153-8

**Published:** 2023-04-06

**Authors:** Moises Alberto Suarez-Zdunek, Sunil Kumar Saini, Christian Ross Pedersen, Sebastian Rask Hamm, Annemette Hald, Allan Rasmussen, Jens Georg Hillingsø, Sine Reker Hadrup, Susanne Dam Nielsen

**Affiliations:** 1 Viro-Immunology Research Unit, Department of Infectious Diseases, Copenhagen University Hospital - Rigshospitalet, Copenhagen, Denmark; 2 Department of Health Technology, Technical University of Denmark, Kongens Lyngby, Denmark; 3 Department of Surgical Gastroenterology, Copenhagen University Hospital - Rigshospitalet, Copenhagen, Denmark; 4 Department of Clinical Medicine, Faculty of Health and Medical Sciences, University of Copenhagen, Copenhagen, Denmark

**Keywords:** Cytomegalovirus infections, Varicella Zoster Virus infection, Liver transplantation, T-Lymphocytes, Inflammation, Immunodominant Epitopes, Major Histocompatibility Complex

## Abstract

**Background:**

Life-long immunosuppressive treatment after liver transplantation (LT) prevents graft rejection but predisposes the LT recipient to infections. Herpesvirus infections are associated with morbidity and mortality among LT recipients. Among those, especially cytomegalovirus (CMV) and varicella-zoster virus (VZV) pose challenges after LT. The aim of this study is to provide an in-depth characterization of the cellular immune response against CMV and VZV infections in LT recipients and identify potential risk factors for infection.

**Methods:**

The Herpesvirus Infections in Solid Organ Transplant Recipients – Liver Transplant Study (HISTORY) consists of an epidemiological and immunological substudy. *The epidemiological substudy* is a retrospective observational cohort study that includes all patients who underwent LT in Denmark between 2010 and 2023 (*N ≈* 500). Using data from nationwide hospital records and national health registries, the incidence of and clinical risk factors for CMV and VZV infections will be determined. *The immunological substudy* is an explorative prospective observational cohort study including patients enlisted for LT in Denmark during a 1.5-year period (*N* > 80). Participants will be followed with scheduled blood samples until 12 months after LT. CMV- and VZV-derived peptides will be predicted for their likelihood to be presented in participants based on their HLA type. Peptide-MHC complexes (pMHC) will be produced to isolate CMV- and VZV-specific T cells from peripheral blood mononuclear cells before and after CMV and VZV infection. Their frequency, T cell receptor sequences, and phenotypic characteristics will be examined, and in a subset of participants, CMV- and VZV-specific T cells will be expanded ex vivo.

**Discussion:**

This study will provide novel insight into T cell immunity required for viral control of CMV and VZV and has the potential to develop a prediction model to identify LT recipients at high risk for infection based on a combination of clinical and immunological data. Furthermore, this study has the potential to provide proof-of-concept for adoptive T cell therapy against CMV and VZV. Combined, this study has the potential to reduce the burden and consequence of CMV and VZV infections and improve health and survival in LT recipients.

**Trial registration:**

ClinicalTrials.gov (NCT05532540), registered 8 September 2022.

**Supplementary Information:**

The online version contains supplementary material available at 10.1186/s12879-023-08153-8.

## Background

Liver transplantation is the only curative treatment of end-stage liver disease, and each year, around 60 patients undergo liver transplantation at Copenhagen University Hospital – Rigshospitalet, which is the only liver transplantation center in Denmark [1]. Life-long immunosuppressive treatment after liver transplantation is necessary to prevent graft rejection, and calcineurin inhibitors and mycophenolic acid that target the T cell-mediated immune responses are the first choice of therapy.

*Herpesviridae* is a family of DNA viruses commonly known as herpesviruses which include, among others, cytomegalovirus (CMV) and varicella-zoster virus (VZV). Infection with herpesviruses is widespread, and > 90% of adults have been infected with at least one of these viruses. It is characteristic for herpesviruses that a transient primary infection is followed by a latent form that can reactivate, especially during periods of immunosuppression. T cells play a pivotal role in the immune response to herpesviruses and are essential for protection and effective viral clearance. Therefore, infections caused by herpesviruses are very common in organ transplant recipients, and especially CMV and VZV infections are major challenges post-transplantation.

CMV is the most common opportunistic infection post-transplantation, it causes significant morbidity and mortality and is associated with loss of the graft [2, 3]. CMV infections are often caused by reactivation of latent CMV infection and have an incidence of around 40% within one year after liver transplantation [4]. VZV infection, most commonly as herpes zoster, is the second-most common infection in solid organ transplant recipients. A meta-analysis found the annual incidence of herpes zoster to be around 9% in liver transplant recipients [5]. Solid organ transplant recipients affected by herpes zoster have an increased risk of disseminated disease and post-herpetic neuralgia compared to immunocompetent controls [6, 7].

Although impaired T cell immunity plays a major role in herpesvirus infections, the antigen-specificity and long-term function of herpesvirus specific T cells have not been evaluated in liver transplant recipients. Increased knowledge of the immune response against CMV and VZV may help identify high-risk liver transplant recipients, and knowledge of the protective immune response may provide insight into and ideas for new treatment modalities against CMV and VZV infections in liver transplant recipients and other immunosuppressed populations.

### Study objectives

The overall aim of this study is to investigate the immune response against CMV and VZV infections in liver transplant recipients and identify potential risk factors for infection.

#### AIM 1

To determine the impact of the level of immunosuppression on the frequency of CMV- and VZV-specific T cells in liver transplant recipients.

#### AIM 2

To provide detailed clinical and in-depth characterization of CMV- and VZV-specific T cell immunity of liver transplant recipients with and without CMV and VZV infection after liver transplantation.

#### AIM 3

To identify independent risk factors for CMV and VZV infection in liver transplant recipients, to identify individuals with high risk of infection, and to create an algorithm for a personalized prevention.

#### AIM 4

To determine the characteristics of a protective immune response against CMV and VZV infections, including epitopes recognized within the virus and the functional and phenotypic characteristics of a protective T cell immune response.

## Methods

The Herpesvirus Infections in Solid Organ Transplant Recipients – Liver Transplant Study (HISTORY) is a retrospective and prospective cohort study that consists of an epidemiological and an immunological substudy. The study is a collaboration between clinicians at the Copenhagen University Hospital – Rigshospitalet who are responsible for recruitment and follow-ups and translational immunologists at the Technical University of Denmark (DTU) who are responsible for the immunological analyses.

### Epidemiological substudy

#### Overview

The epidemiological substudy is a retrospective observational cohort study investigating the incidence and clinical risk factors for CMV and VZV infections in all patients who underwent liver transplantation in Denmark between 1 and 2010 and 1 January 2023.

#### Eligibility criteria

All persons in Denmark over the age of 18 who underwent liver transplantation at Copenhagen University Hospital – Rigshospitalet will be eligible for inclusion.

#### Clinical data

In Denmark, all hospital records since 1 December 2010 are accessible through a nationwide digital platform. From hospital records, we will collect clinical data regarding demographics, medical and surgical history, biochemistry, microbiology, including donor and recipient herpesvirus serology at the time of transplantation as well as recipient PCR and serology after liver transplantation, pathology results, and radiology descriptions.

Furthermore, data will be retrieved from The National Danish Patient Register (NPR), the Danish Cause of Death Register (DAR), and the Danish National Prescription Registry (LSR). From NPR, which contains data on hospital admissions since 1994, including discharge diagnoses using the ICD-10 classification [8], we will collect data on admissions due to CMV and VZV infections. From DAR, which contains the dates and causes of death since 1970 in Denmark [9], we will collect data on participant mortality and causes of death. From LSR, which contains data on sales of prescription-only drugs since 1994 [10], we will collect data on the use of drugs for treatment of infections.

### Immunological substudy

#### Overview

The immunological substudy is a prospective observational cohort study in which patients enlisted for liver transplantation in Denmark will be invited to participate from 1 to 2023 until *N* > minimum 80 or until 1 January 2026, whichever comes first. Participants will be included from the liver transplantation waitlist and will be followed with scheduled blood samples until 12 months after liver transplantation and with searches in hospital records and national health registries until 1 January 2033.

#### Eligibility criteria

All persons in Denmark over the age of 18 enlisted for liver transplantation at Copenhagen University Hospital – Rigshospitalet will be eligible for inclusion. Persons who do not understand the provided information regarding the study will be excluded.

#### Blood sampling

Blood samples will be collected from participants at scheduled visits. All included participants will provide a 50 mL blood sample at the time of inclusion prior to liver transplantation. Participants will also be invited to repeated blood samples 1, 3, 6, and 12 months after liver transplantation, as well as after primary infection and/or reactivation of CMV and/or VZV. The samples will be collected in lithium-heparin and EDTA-coated collection tubes.

Within 4 h after collection, blood collected in lithium-heparin-coated collection tubes will be transported to the laboratory for purification of peripheral blood mononuclear cells (PBMC) through density gradient centrifugation. EDTA-coated collection tubes will be stored on ice immediately after collection and transported to the laboratory for centrifugation within 1 h, after which plasma will be separated and stored at -80 °C. All PBMC and plasma will be stored at a biobank at the Department of Health Technology, DTU. PBMC will be cryogenically preserved in liquid nitrogen.

#### Questionnaire

At the time of inclusion, participants will fill out a questionnaire regarding health, lifestyle, socioeconomic status, use of medicines, and previous vaccinations. The questionnaire will be entered directly into a secured digital platform or on paper (see Additional file 1).

#### Identification of herpesvirus-specific T cells

**Fig. 1:**
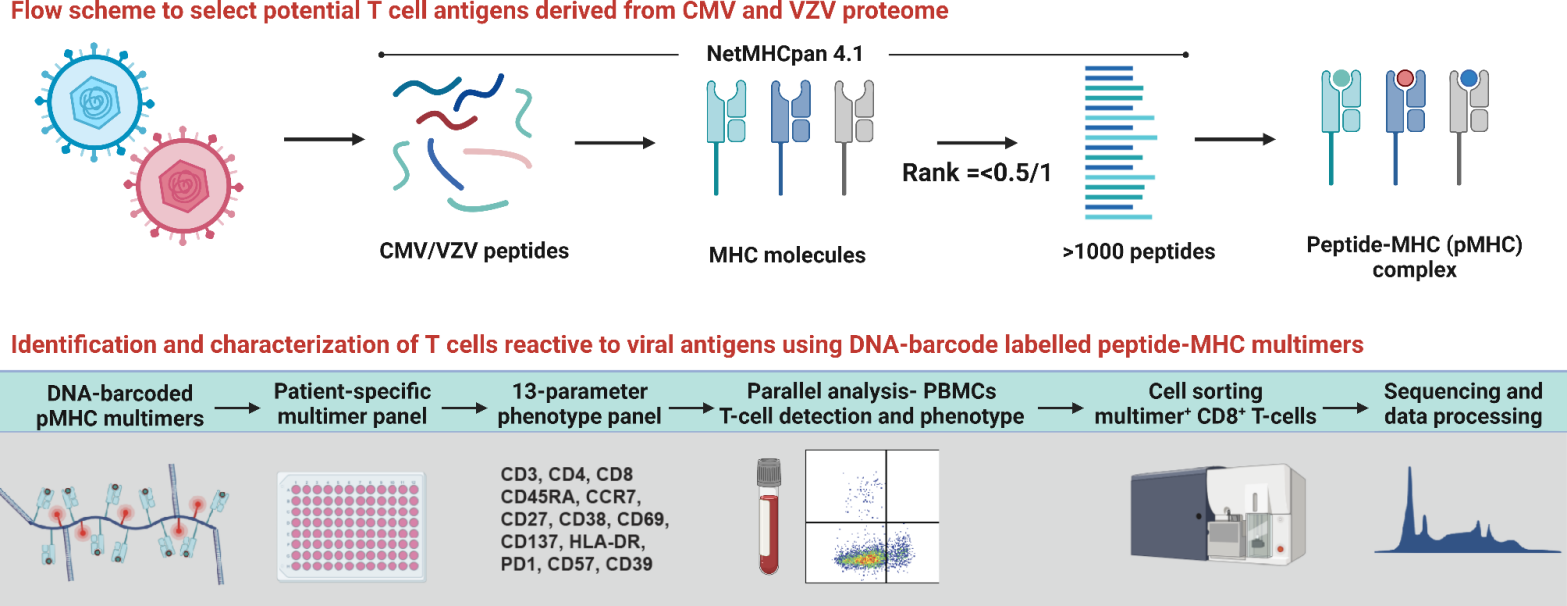
**Overview of methods for identification and evaluation of T cells using peripheral blood mononuclear cells.** Using the NetMHCpan 4.1 platform, potential cytomegalovirus (CMV) and varicella-zoster virus (VZV) peptide-MHC (pMHC) complexes will be predicted corresponding to the most frequent HLA haplotypes amongst participants. The pMHC complexes will be combined in a DNA-barcoded patient-specific pMHC multimer. Peripheral blood mononuclear cells (PBMCs) will be examined for the presence of pMHC multimer-binding T cells and sorted using flow cytometry to examine their antigen recognition profile, as well as their expression of phenotypic cell surface markers. The reactive T cells may be further examined for their frequency, T cell receptor sequence, and potential for ex vivo expansion

To identify the complete repertoire of CMV- and VZV-specific T cells and their immunodominance in CMV and VZV infection, respectively, we will perform a broad T cell screening towards all potential epitopes. Human leukocyte antigen (HLA)-class I presented epitopes will be predicted using NetMHCpan-4.1 and synthetized to make peptide-HLA multimers matching the HLA profile of the participant [11] (Fig. 1). We will cover the 12 most frequent HLA haplotypes. Using DNA-barcode labeled peptide-MHC multimers and subsequent flow cytometry-based sorting of CMV- and VZV-specific T cells, we can determine the T cell recognition of a large library of potential epitopes (> 1000/sample) [12, 13]. The sequential sampling pre- and post-transplantation will allow for a comparative assessment and monitoring of T cell frequency at different levels of immunosuppression.

#### Phenotype and cytokine profiling

**Fig. 2:**
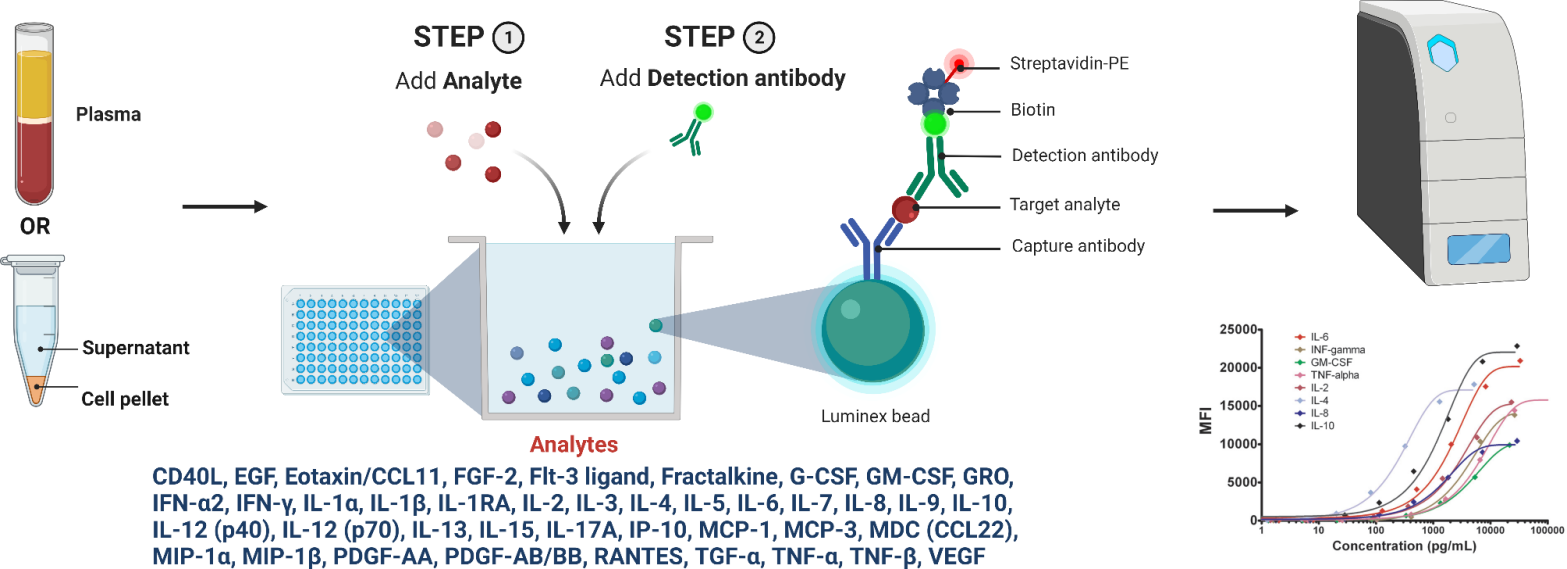
**Overview of methods for cytokine profiling using Luminex MILLIPLEX® assay.** Plasma contains an unspecified concentration of analytes of interest, i.e., cytokines, chemokines, interleukins, and growth factors. To determine the concentrations of the analytes, each Luminex well contains multiple types of color-coded beads conjugated to a capture antibody that binds the specific analyte of interest. In presence of the analyte, a biotin-conjugated detection antibody will bind to the capture antibody-analyte complex. Adding phycoerythrin-labeled streptavidin will produce fluorescence that is color-specific for each analyte and allows for measuring the mean fluorescence intensity (MFI) of each analyte in each Luminex well. Comparing MFI at different dilution levels with standard solutions allows for determining the concentrations of the analytes of interest

Phenotyping and functional characterization of specific T cell populations towards immunodominant epitopes in VZV and CMV before and after liver transplantation will be carried out using a flow cytometry panel of 13 cell surface and intracellular markers of activation, senescence, and exhaustion (Fig. 1). Furthermore, we will measure the cytokine profiles in plasma samples using an established Luminex MILLIPLEX assay to simultaneously quantify 41 analytes that map the complete cytokine signature including interleukins, chemokines, and growth factors (Fig. 2).

#### T cell receptor sequencing

We have recently developed a technology that allows for collection of large sets of T cell receptor (TCR) sequences paired to different peptide targets based on their pMHC recognition motif through a pMHC multimer linked to a DNA barcode [14]. Using single-cell capture and analysis systems such as 10x genomics based on sorted pMHC responsive T cells, we can pair the TCR sequences and the DNA barcode, assigning the pMHC specificity. Hereby, we provide a high-throughput platform for identification of TCR-pMHC pairs. Using this platform, we will identify T cell clonotypes and describe their functional characteristics. We will implement parallel phenotyping of immune cells using a 150 + marker panel of DNA barcoded antibodies that targets a broad array of lineage markers, activation markers, exhaustion markers, and regulatory markers.

#### T cell expansion for proof-of-concept of adoptive T cell transfer

We have established a new technology to expand viral antigen-specific T cells ex vivo that uses artificial antigen-presenting cells (aAPC) scaffold-based stimulation of T cells to create the ideal specificity and functionality (manuscript in draft). These aAPC scaffolds are interconvertible allowing a personalized design. We will use this method to expand T cells specific to selected immunodominant VZV and CMV epitopes from pre-liver transplantation samples from 5 participants.

#### Clinical data

Clinical data will be collected from patient records and health registries as described previously for the epidemiological substudy.

### Statistics

#### Sample size

Due to the explorative nature of the immunological substudy, formal power calculations cannot be conducted. Based on experience from another study on T cell reactivity in patients with COVID-19, a sample of 60 participants, of whom 20 were infected, was sufficient to determine the characteristics of an effective immune response [12]. We expect to include 80 participants to allow for some heterogeneity in baseline characteristics. With an incidence of CMV of around 40% within a year after liver transplantation, around 20–30 participants are expected to develop active CMV infection within 1 year after liver transplantation [4], which meets the requirements from the previous study. With an incidence of VZV of 9% each year, around 10 participants are expected to develop active VZV within the period [5], but as in this study, participants act as their own controls, we expect to increase the power sufficiently to enable us to meet the aims.

#### Planned statistical analyses

Continuous data will be compared using Student’s *t*-or Mann–Whitney *U*-tests, and categorical data using Pearson’s χ^2^ or Fisher’s exact tests, as appropriate. For the epidemiological substudy, cumulative incidences of CMV or VZV infections will be determined by the Aalen-Johansen estimator, with death and re-transplantation as competing risks. Risk factors for CMV or VZV infections will be investigated using time-updated multivariate Cox proportional-hazards regression models adjusting for age and sex in the base model. For the prediction model including clinical risk factors for infection, each risk factor will first be assessed in univariable models, and then risk factors with a *P* < 0.1 will be included in a multivariable model. The model will be evaluated in a validation cohort derived from Scandiatransplant. Finally, immunological markers will be included in the model, and a prediction algorithm will be developed in collaboration with the biostatistical team at DTU.

## Discussion

Limited treatment options are available for CMV and VZV infection. Ganciclovir, the drug of choice for CMV, carries a risk of serious side effects, and resistance is an emerging problem [15]. To avoid CMV infection in liver transplant recipients, routine programs for prophylaxis with valganciclovir have been implemented. However, due to toxicity, new treatment options are warranted. Although impaired T cell immunity plays a major role in herpesvirus infections, antigen-specificity and long-term functional state of these T cells have not been evaluated in liver transplant recipients. By identifying T cell clonotypes and their functional characteristics, this study has the potential to evaluate immunological risk factors for CMV and VZV in liver transplant recipients. By identifying clinical risk factors for CMV and VZV infections in liver transplant recipients, this study has the potential to develop a prediction model including both clinical and immunological risk factors to identify liver transplant recipients at high risk for infection, which may pave the road for a personalized approach to prevention of CMV- and VZV-related complications after liver transplantation.

Adoptive T cell therapy (ACT) has been successfully explored in hematopoietic stem cell transplantations [16, 17]. A crucial pre-requisite for optimal ACT is the generation of sufficient numbers of antigen-specific T cells. The expansion of T cells specific to selected immunodominant CMV and VZV epitopes planned for a subset of participants in this study has the potential to generate a proof-of-concept for an ACT platform that could form the basis for future clinical applications in patients where existing treatment is contraindicated.

## Electronic supplementary material

Below is the link to the electronic supplementary material.

Supplementary Material 1

## Data Availability

The datasets generated and/or analyzed during the current study are not publicly available as they contain information that could compromise participant privacy and consent but are available from the corresponding author on reasonable request and with permission of S.D. Nielsen and S.R. Hadrup.

## List of abbreviations

HISTORY: Herpesvirus Immunology in Solid Organ Transplant Recipients - Liver Transplant Study
LT: Liver transplantation
CMV: Cytomegalovirus
VZV: Varicella-zoster virus
MHC: Major histocompatibility complex
pMHC: peptide-major histocompatibility complex
DTU: Technical University of Denmark
PCR: Polymerase chain reaction
NPR: The National Danish Patient Register
DAR: Danish Cause of Death Register
LSR: Danish National Prescription Registry
ICD-10: International Classification of Diseases version 10
EDTA: Ethylenediaminetetraacetic acid
PBMC: peripheral blood mononuclear cells
HLA: human leukocyte antigen
TCR: T cell receptor
aAPC: artificial antigen-presenting cell
COVID-19: Coronavirus disease 2019
ACT: Adoptive T cell therapy
