# Supplementary material for: Herpesvirus immunology in solid organ transplant recipients – liver transplant study (HISTORY): a retrospective and prospective observational cohort study
Source: BMC Infect Dis. 2023 Apr 6;23:214. doi: 10.1186/s12879-023-08153-8 (PMC10078045; doi:10.1186/s12879-023-08153-8)
Supplement: Supplementary file 1 — Supplementary Material 1 [file 12879_2023_8153_MOESM1_ESM.pdf]

HISTORY-ID nr.: ___________________
(filled out by study personnel)

HISTORY STUDY
QUESTIONNAIRE

In this questionnaire we will ask you to answer some questions about your health and lifestyle. **We ask you to answer all questions**. The questions are answered by ticking the box that is most appropriate. Naturally, your answers will be treated with **strict confidentiality**.

Name			
Address			
Postal code & city			
Telephone no.		CPR no.	
E-mail			

Vaccination history

In the following we ask you only to state vaccinations that you have either:

1. Received abroad before or after November 15, 2015, *or*
2. Received in Denmark before November 15, 2015

Vaccinations that do not fulfil the two criteria are to be omitted.

For vaccines given in series, please state the year of completing the series.

EXAMPLE: Vaccination against hepatitis A+B is often provided in a series of 3 doses. In such case, provide the year of the third dose. Influenza vaccines are often given once a year, but they do not constitute one series, and the year is provided for each vaccination.

	Yes	No	Un-known	Approx. year(s):	If known: What type of vaccine?
Tuberculosis	☐	☐	☐	_______	_______________
Human papillomavirus (HPV)	☐	☐	☐	_______	_______________
Haemophilus influenzae type B (Hib)	☐	☐	☐	_______	_______________
Chickenpox/shingles (varicella-zoster)	☐	☐	☐	_______	_______________
Hepatitis A	☐	☐	☐	_______	_______________
Hepatitis B	☐	☐	☐	_______	_______________
Influenza (1st time)	☐	☐	☐	_______	_______________
“ (2nd time)	☐	☐	☐	_______	_______________
“ (3rd time)	☐	☐	☐	_______	_______________
“ (More than 3)	☐	☐	☐	_______	_______________
Pneumococcal disease (incl. pneumonia)	☐	☐	☐	_______	_______________

Health and lifestyle questionnaire

In the following, we kindly ask you to respond to questions regarding your lifestyle and health. Please respond to the best of your ability. If you do not know the exact year, please provide the best approximate.

	Question	Yes	No
1.	Do you experience pain or tightness in your chest when you are in a hurry, or when you use stairs?	☐	☐
2.	Do you have shortness of breath when you are in a hurry or go up a hill?	☐	☐
3.	Do you have more shortness of breath when walking at normal pace on a straight road compared to people your own age?	☐	☐
4.	Do you sometimes have to stop and catch your breath when walking down the street at your own pace?	☐	☐
5.	Do you sometimes wake up at night due to shortness of breath or strenuous breathing?	☐	☐
6.	Do you have shortness of breath when taking a bath or when getting dressed?	☐	☐
7.	Do you often experience shortness of breath when you wake up in the morning?	☐	☐
8.	Do you have shortness of breath when sitting quietly or resting?	☐	☐
9.	Have you coughed persistently for the past 8 weeks?	☐	☐
10.	Do you cough up mucus (in the mornings or during the day) as long as 3 consecutive months a year?	☐	☐
	**If yes:** Has this been going on for 2 consecutive years or more?	☐	☐
11.	Have you been told by a doctor or nurse that you have COPD (also called smoker lungs)?	☐	☐
	**If yes:** How old were you when you got diagnosed with COPD? ___years		
12.	Have you been exposed to dust or fumes over long periods of time in your job?	☐	☐
	**If yes:** How many years? Number of years: ______		
13.	Do you sometimes experience wheezing (highpitched whistling sound during breathing)?	☐	☐
	**If yes:** During a cold?	☐	☐
	During physical activity?	☐	☐
	Without known cause?	☐	☐
21.	Have you had an episode of acute fever in the last four weeks?	☐	☐
24.	Do you smoke?	☐	☐

If no: Have you previously smoked? ☐ ☐

If you have never smoked, please go to question 29.

25. How many years have you smoked? Number of years _____

26. How old were you when you started smoking?_________years

27. **If you used to be a smoker**, how old were you when you stopped smoking?_______years

28. If you smoke or have previously smoked, how much is/was your average consumption of:

Cigarettes without filter	Quantity per Day: __
Cigarettes with filter	Quantity per Day: __
Cheroots	Quantity per Day: __
Cigars	Quantity per Day: __
Pipe tobacco	Packets of 40/50 g per week: _______

34. How often do you drink alcohol? Number of units **per week**: _______

46.	Indicate your physical activity AT WORK in the last year (also filled in by housewives, students and currently unemployed, while pensioners without actual employment are asked to go to question 47.) **(Only one answer)**

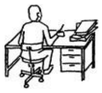

I. Predominantly sitting work
e.g., desk job, homemaker without children, and with a maid ☐

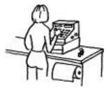

II. Sitting or standing, sometimes walking
e.g., clerk, teacher, homemaker who does all the washing and cleaning themselves, without small children ☐

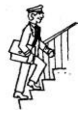

III. Walking, sometimes lifting
e.g., postman, health care worker, who does all the washing and cleaning themselves, with one or more small children ☐

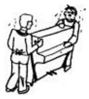

IV. Heavy bodywork
e.g., movers, construction workers ☐

If yes to III or IV: Do you often lift heavy loads? Yes ☐ No ☐

On average, how many hours a week do you work outdoors? Hours___________

47.	Indicate your physical activity DURIN LEISURE TIME (including transport to and from work) within the last year **(Only one answer)**

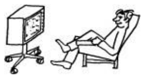

I. Almost completely physically inactive or light physical activity up to 2 hours a week.
e.g., reading, television, cinema ☐

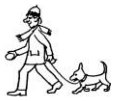

II. Light physical activity from 2 - 4 hours a week
e.g., walks, biking, light gardening, light exercise ☐

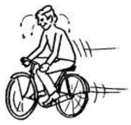

III. Light physical activity for more than 4 hours per week or more intense physical activity for 2 - 4 hours per week
e.g. fast walking and/or fast cycling, laborious gardening, heavy exercise with sweating or breathlessness ☐

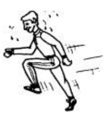

IV. Intense physical activity for more than 4 hours a week or regular intense training and potentially with participation in competitions several times a week ☐

If yes to III or IV: Does your training involve weight-lifting or heavier strength/weight?

Yes ☐ No ☐

On average, how many hours per week have you been outdoors in your spare time?
Hours: _______

Socioeconomic questionnaire

In the following, we kindly ask you to respond to questions regarding your education and demographics.

48. How long is your education?
(from primary to upper secondary education) (max. 14 years): Years: ______

49. What is the longest education you have completed <u>after</u> you left primary school? (**one X**)
☐No education
☐Short-cycle higher education (e.g., academy of professional higher education)
☐Vocational training or similar (1-3 years)
☐Medium-cycle higher education (e.g., teacher, nurse etc.)
☐Long-cycle higher education
☐Ph.d. and equivalent

53. Do you live:
☐ With spouse/companion
☐ Alone
☐ With others

54. How many people are in your household including yourself? Quantity: _____
